# The Sufferings of Christ

**Published:** 1887-11-12

**Authors:** 


### WORDS OF CONSOLATION.

[Communications for this column should be addressed "Chaplain," care of the Editor of THE HOSPITAL, who will gratefully welcome any suggestions or inquiries from matrons, nurses, and the staff generally.]

### LVIII.—THE SUFFERINGS OF CHRIST.

*(For Reading to the Sick.)*

IN taking to heart the details of our Lord's Passion we shall do well to remember His bonds. Surely if St. Paul could say, "Remember my bonds," how much more can Christ call upon us in the same words. He was bound when first arrested. Annas sent Him bound to Caiaphas. He was bound for the scourging. Some think that He was bound, as well as nailed, to the Cross; but this is uncertain. Through the Passion He was suffering from the pressure of strong cords. May not all this point to the remedy for abused liberty? May we not see here the atonement for sins committed in the much-vaunted name of liberty? How often when the first sense of freedom breaks in upon young hearts, as they begin to think and act for themselves, there comes also the abuse of it. And how often the abuse of liberty consists in the members of the body being given to unrighteousness. Because they are free they become slaves. Liberty first of all descends to license, and license degenerates into slavery. So do many who begin by boasting of being able to do as they like, or at least by casting aside the cords of true discipline and self-restraint, find themselves tied and bound with the chain of their sins. What, then, is the remedy for this abuse of liberty? Repentance! Yes, but repentance strictly based upon the lines so plainly indicated by the suffering Christ. The sin is atoned for, but the sinner can only regain his freedom by a persevering, diligent, and complete curbing of the members, or it may be one member in particular, once given to unrighteousness. The tongue given to so-called free or filthy conversation must be restrained. The hands which have lent themselves to dishonest practices must never be stretched forth again in such a service. In every case of sin through the members, if amendment be sincere, that one through which the great offence has come will be tied and bound in such a sense as to go and sin no more. In this way, and this only, can true freedom be regained. Perfect freedom, the glorious liberty of the children of God, can only be regained by those of us who have once trifled with the supreme law of liberty, through persevering self-restraint. Otherwise, what is the punishment pointed to hereafter? What is the retribution for abused liberty? "Bind him hand and foot and take him away, and cast him into outer darkness. There shall be weeping and gnashing of teeth" (Matt. xxii. 13). "Bind them in bundles for burning" (Matt. xiii. 30). Captivity is a very common expression in Holy Scripture for the condition of people who are under the dominion of sin, while sin in its very earliest step is the abuse of liberty, the perversion of free-will. There, then, we may discern most cogent reasons for our Lord's bonds being so soon fastened upon Him, and for His being so grievously afflicted with cords throughout the Passion. This is why we find bondage so vividly depicted as a condition of the lost. So the Passion provides the sacrifice and the remedy for sin, and points to the inevitable retribution if the former be despised. "O God, who art the author of peace and lover of concord, in knowledge of whom standeth our eternal life, whose service is perfect freedom. Defend us, Thy humble servants, in all assaults of our enemies."

*(To be continued.)*

